# An Animal Ex Vivo Model Comparing Two Different Methods of Sinus Floor Elevation with Great Elevation Heights: Macroscopic, Microscopic and Radiological Analysis

**DOI:** 10.3390/dj12110337

**Published:** 2024-10-22

**Authors:** Erick Rafael Fernández Castellano, Lorena Benito Garzón, Magaly Teresa Márquez Sánchez, Javier Flores-Fraile

**Affiliations:** 1 Department of Surgery, University of Salamanca, 37007 Salamanca, Spain; kcire.008@yahoo.es (E.R.F.C.); j.flores@usal.es (J.F.-F.); 2 Department of Human Anatomy and Histology, University of Salamanca, 37007 Salamanca, Spain; lorenabenito@usal.es; 3 Salamanca Biomedical Research Institute, 37007 Salamanca, Spain

**Keywords:** balloon elevation, elevation height, sinus floor elevation, elevated mucosa

## Abstract

Introduction: Maxillary sinus pneumatization increases with age and tooth loss, leading to a reduction in the maxillary alveolar ridge, which often results in insufficient bone height for the proper placement of dental implants. This study focused on performing maxillary sinus elevations in ex vivo bisected pig heads using novel access and elevation devices, comparing these with the osteotome sinus floor elevation (OSFE) technique. Materials and Methods:An experimental study was conducted using 20 ex vivo adult pig heads. The sinus elevations were divided into two groups: 10 heads were treated using the osteotome technique, and 10 heads were treated using a new device, which consists of a syringe with latex and saline solution, as well as a burr system for membrane access and control. Results: In the osteotome technique, perforations of the Schneiderian membrane were observed, whereas the inflatable balloon device did not cause any lacerations. Conclusions: OSFE resulted in sinus membrane perforations at greater elevation heights, while the new balloon device successfully elevated the membrane without tearing it. Within the limitations of this study, maxillary sinus lifts using the new balloon technique proved to be minimally invasive procedures.

## 1. Introduction

The maxillary sinuses are the second most common sources of infection after the ethmoid sinuses. With an average volume of 12.5 mL, they are air-filled spaces located in both jaws. Of the four paranasal sinuses, the maxillary sinus is the oldest. It is also the first to appear on animals and humans. They are located in the body of the maxillary bone just behind the canine and premolars and are surrounded by Schneider’s membrane, which is a thin bilaminar mucoperiosteal membrane formed by a ciliated pseudostratified columnar epithelium on the luminal side and a unicellular osteogenic periosteal layer on the osseous side. The membrane is delimited by the following:

- The Superior wall or roof forms the floor of the orbit and is related to the lacrimal sac.
- The Inferior wall or floor is formed by the alveolar process of the maxilla and the bony palate, and is related to the dental alveoli of the first and second molars.
- The Anterior wall is a facial part covered by the soft tissues of the cheek. It extends from the alveolar process to the inferior orbital rim and from the piriform orifice to the proximities of the body and zygomatic bone.
- The Posterior wall is related to the pterygopalatine fossa and its contents (Internal Maxillary Artery, Pterygopalatine Ganglion, and branches of the Trigeminal Nerve).
- The Bony base is formed above by the ethmoid bone and its unciform process, in front by the lacrimal bone or unguis, below by the inferior turbinate bone and ethmoidal process, and behind by the maxillary process of the palatine [1].

Maxillary sinus pneumatization increases with age and edentulism, leading to a reduction in the maxillary alveolar ridge and resulting in insufficient bone height to support dental implants. Consequently, the sinus floor must be elevated to achieve an acceptable bone volume. Various sinus floor elevation techniques have been developed, including the lateral window approach (Boyne and James, 1980; Tatum, 1986) [2]. The initial approach explained by Tatum involved a combination of incisions that allowed for the reflection of a buccal flap to expose the external bony wall of the sinus, in which a window was made to access the sinus cavity and elevate Schneider’s membrane to introduce a bone graft, including autologous bone, bone substitutes, synthetic biomaterials or combinations of these substances [3]. This approach is often rejected by patients due to cost, fear or other considerations [4,5,6,7,8].

Summers first proposed the transcrestal sinus floor elevation using an osteotome (OSFE) approach [9,10]. The advantages of this procedure over the conventional lateral maxillary sinus elevation approach include reduced trauma, a simpler and less invasive intervention, and therefore, better postoperative outcomes. Lai et al. compared sinus elevation using osteotomes with and without grafting and concluded that there were no significant differences between the two groups in implant survival rates, with cumulative survival rates for both groups being 97.38% and 92.13%, respectively [11]. This method is considered a less invasive, less traumatic and more cost-effective procedure. However, the OSFE procedure carries a higher risk of sinus membrane perforations due to visual limitations [9].

To overcome the limitations of Summers’ OSFE, several devices and systems have been proposed and used, including the balloon technique, as shown in Table 1. The advantage of the balloon technique is that it can be used in the presence of residual bone of 3 mm or more [12], whereas conventional transcrestal elevation with osteotomes requires a minimum of 6 mm of residual crestal bone [7].

Muronoi et al., in 2003 [13], and Soltan et al., in 2005 [14], described the use of the sinus balloon in direct sinus elevation by placing it through a window in the lateral sinus wall. Kfir et al., in 2006 [15], described transcrestal sinus elevation using the sinus balloon technique, with bone grafts and dental implants placed in the same surgical procedure.

This study focused on comparing maxillary sinus elevations in ex vivo bisected pig heads using the author’s patented new access and elevation devices with the osteotome technique. The objective was to evaluate these indirect sinus floor elevation methods macroscopically, microscopically and radiographically. We assume that the null hypothesis is that the new device will perforate Schneider’s membrane similarly to the osteotome technique, at elevations greater than 4 mm in height.

**Table 1:** Review of the literature on indirect elevation heights.

Authors	Elevation Method	Design	Elevation Height (mm)
Summers (1994) [2]	OSFE	Technical report	4–5
Summers (1994) [2]	BAOSFE	Case report	5–7
Zitzmann and Scharer (1998) [5]	OSFE	Clinical study	4–5
Rosen et al. (1999) [8]	BAOFE	Clinical study	5–7
Baumann and Ewers (1999) [16]	ECOSFE	Experimental study	7–10
Ioannidou and Dean (2000) [6]	OSFE	Case report	4–5
Nkenke et al. (2002) [17]	ECOSFE	Clinical study	2–5
Emmerich et al. (2005) [7]	OSFE	Meta-analysis	1–7
Sotirakis and Gonshor (2005) [18]	Free fluid pressure	Case report	6–9
Benner et al. (2005) [19]	BLC	Technical report	10
Kfir et al. (2007) [12]	Balloon elevation	Case report	10
María Peñarrocha et al. (2007) [20]	Balloon elevation	Case report	8.7
Hadar et al. (2014) [9]	IRAISE	Clinical trial	6.7–13.1
Parthasaradhi et al. (2015) [21]	Sinus lift system	Clinical trial	5.80–10.20
Xian et al. (2017) [22]	SCA KIT	Clinal study	2.8–7.4
Jing Yang et al. (2018) [10]	OSFE	Retrospective study	0.1–8.6
Aditi et al. (2019) [23]	OSFE	Clinical trial	6.99–7.10
Haushu et al. (2020) [24]	IRAISE	Retrospective cohort study	8–9.3

OSFE, osteotome sinus floor elevation; BAOSFE, bone-added osteotome sinus floor elevation; ECOSFE, endoscopically controlled sinus floor elevation; BLC, balloon lift control sinus floor elevation; SCA, sinus crestal approach. iRaise Minimally Invasive Sinus Lift Implant device.

## 2. Materials and Methods

An experimental study was carried out by the same operator using twenty ex vivo adult pig heads. The pig model is well-established in implant research due to its soft tissue covering, which is comparable to that of humans. An ethical approval from the university’s Research Ethics Committee was not required for this study since the heads were purchased as a base material for food processing from a local butcher shop.

Due to the pronounced height of the alveolar crest in adult pigs, a maxillary sinus approach through the lateral wall was preferred for the experiments. The lateral sinus approach provided better control during the dissection of the membrane.

All animals were free from local or systemic diseases to prevent any bias caused by pathological tissue alterations. The experiments were carried out within a standardized maximum timeframe of 6 h postmortem, under a constant ambient temperature of 21 °C.

### 2.1. Elevation Methods

#### 2.1.1. Sinus Floor Elevation with BLC

**Figure 1:**
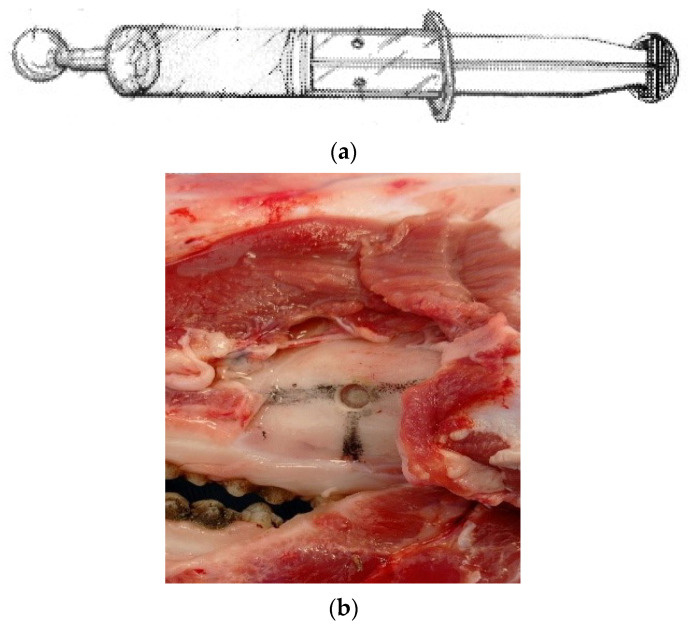

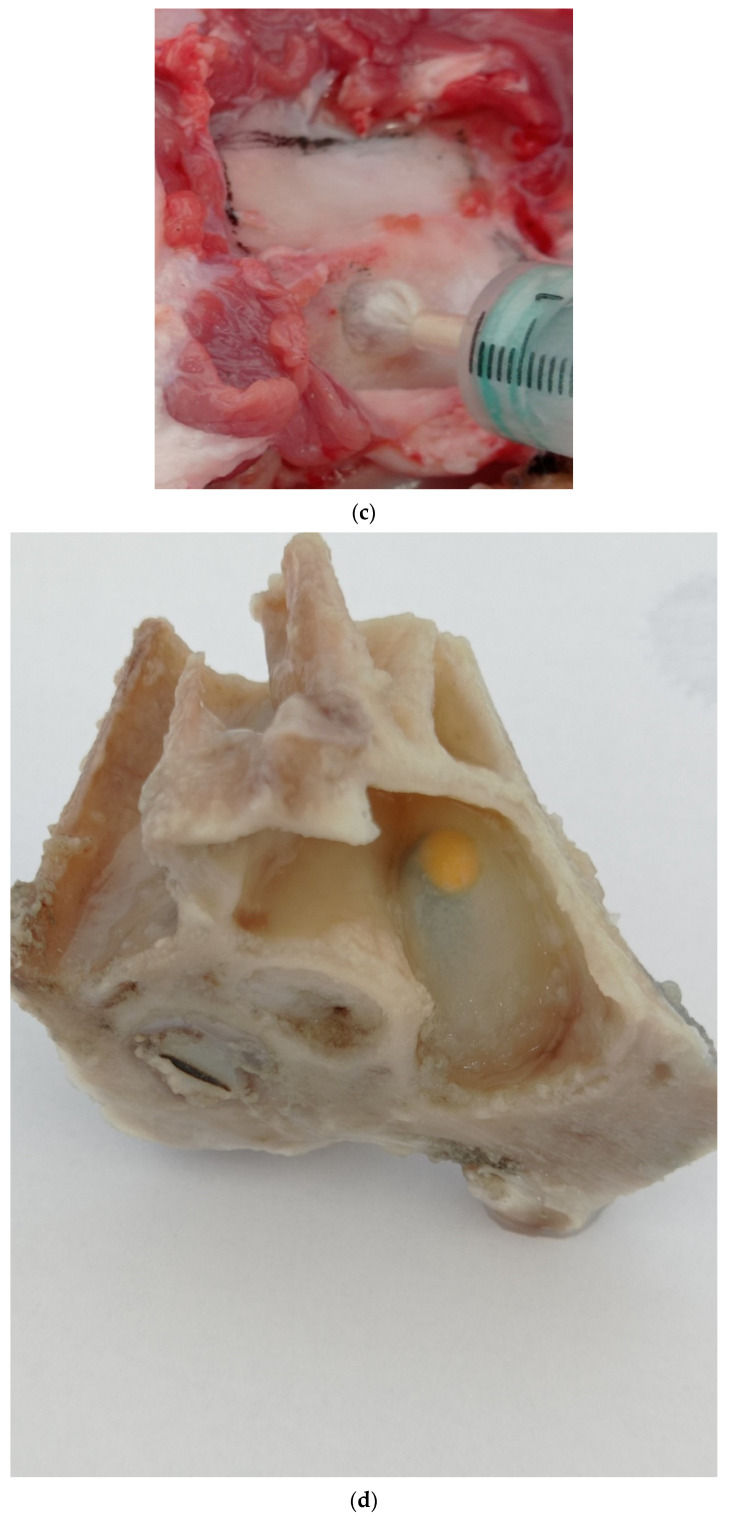

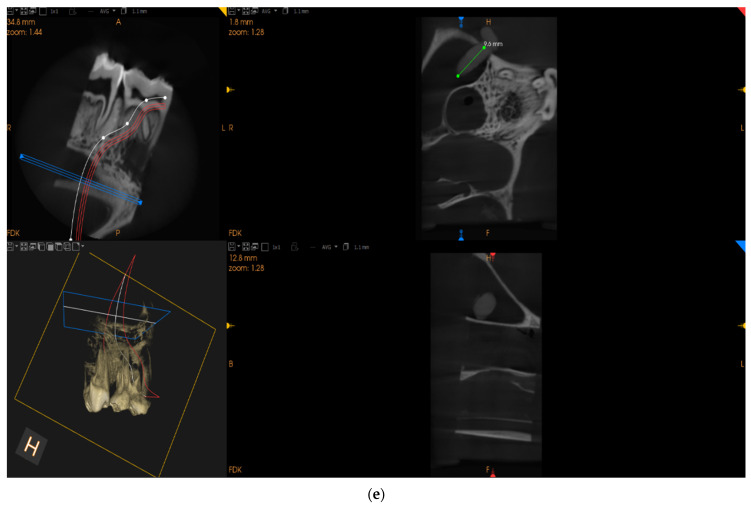
(**a**) New Lifting Device Invented by the Author. (**b**) Elevation Window. (**c**) New Elevation Device and Maxillary Sinus Window OSFE. (**d**) Section for Histological Analysis. (**e**) CBCT 5 × 5 of the Sections Where Achieved Heights Were Measured in the Pig Heads.

The sinus floor elevation was performed using the author’s patented new elevation control system, with international publication numbers WO/2022/200650 and PCT/ES2022/000008 in PATENTSCOPE. This system consists of a syringe with stops, retaining elements, latex and saline solution (see Figure 1a).

To access the sinus, a Bien-Air turbine with a 3.64 mm diameter diamond ball bur was used to mark the window, with ample irrigation. This was followed by the use of a cylindrical diamond bur with smooth lateral walls, which was also designed and patented by the author, with a registration number in the Spanish Industrial Property Bulletin ES1287694U. This bur was attached to a NSK contra-angle handpiece with a 20:1 reduction at 1600 rpm and 35 Ncm torque, powered by an NSK SURGYC PRO motor. Cooling was provided by a saline solution chilled to 3 °C.

The access was made by entering 2 cm perpendicularly to the gingival margin of the first molar (see Figure 1b).

Each elevation was performed within a 3 min timeframe while applying constant pressure to allow the tissue to adapt to the balloon’s tension and separate from the bone surface. Following the insertion into the maxillary sinus, as described above, the balloon was progressively filled to a target volume of 2 mL of serum while pressure was applied. The mean pressure required to elevate the maxillary bone floor mucosa was 344.46 mmHg. The balloon was then deflated, and the device was removed. The membranes were macroscopically inspected to ensure there were no perforations (see Figure 1c).

To access the sinus, an NSK SURGYC PRO motor was used in combination with a surgical handpiece (MICRO-MEGA). The handpiece provided a 20:1 reduction, and the motor speed was set to 1600 rpm. Cooling was provided by a saline solution chilled to 3 °C. For the osteotomy of the internal portion of the cortical bone and the elevation of the mucosa, a conical osteotome with a blunt tip measuring 4.3 mm in diameter was used, which was struck with a mallet until access to the sinus interior was achieved.

The elevation heights with both devices were determined by carefully stretching the mucosa and introducing an impression material (Elite HD+ Putty Soft Regular Set, Zhermack, Badia Polesine, Italy) into the space beneath the elevated mucosa. These were then dissected and cut into approximately 4 cm × 4 cm × 4 cm sections using a jigsaw for histological study, ensuring a sufficient safety margin to avoid accidental damage to the mucosa (see Figure 1d).

Subsequently, CBCT scans with a 5 cm × 5 cm field of view were performed using a CS 8100 3D system to measure the achieved heights in the head samples (see Figure 1e).

#### 2.1.2. Histological Processing of Non-Decalcified Samples Embedded in Plastic

Histological analysis was performed to evaluate microscopically whether all membrane layers were elevated and whether perforation was present with either technique.

The procedure was carried out as follows:

1. **Fixing and Preparation**: Bone samples were collected, preserved in formalin, washed and then dehydrated using a series of ethanol solutions (from 70% to absolute ethanol).
2. **Embedding in PMMA**: The samples were embedded in liquid polymethylmethacrylate (PMMA), a process that took 15 days at 4 °C with agitation, followed by polymerization in glass cylinders for 5–6 days at 32 °C.
3. **Sectioning**: After polymerization, the bone samples were sectioned using a microtome. Initial thicker sections (30 µm) were made until the area of interest was reached, and then finer sections (5 µm) were prepared.
4. **Staining**: The sections were stained using **Goldner’s Trichrome**, a method that highlights the difference between osteoid (unmineralized bone) and mineralized bone, as well as providing insights into the cell morphology.
5. **Microscopic Analysis**: The stained sections were analyzed using a Nikon digital camera Sight DS-SMC attached to a Nikon Eclipse 90i optical microscope.

## 3. Results

A total of 20 CBCT scans with a 5 × 5 cm field of view were performed using a CS 8100 3D system. The length of the achieved elevation was measured (see Table 2), with a mean elevation of 10.26 mm, standard deviation ±1.23, median of 10.10 and a range of 8.00–13.30 mm.

**Figure 2:**
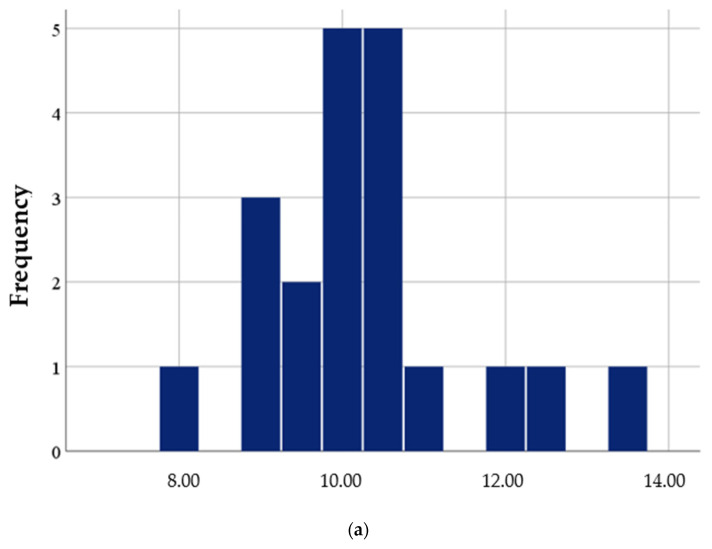

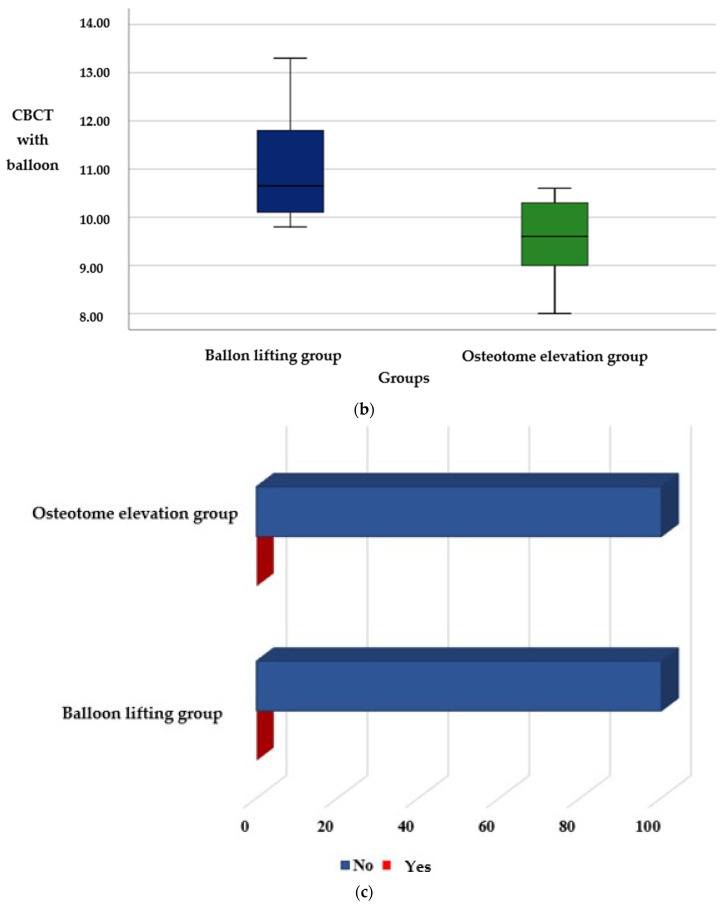

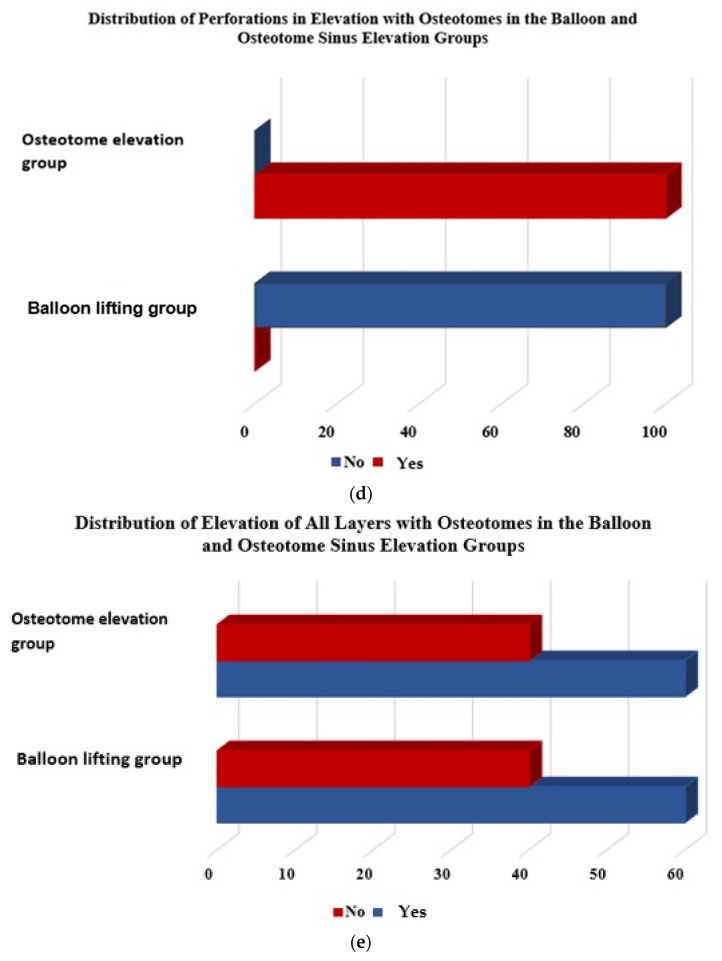
(**a**) Distribution of Elevation Distance from the Crest Measured with CBCT in the Entire Sample. (**b**) Distribution of Elevation Distance from the Crest Measured with CBCT in the Balloon and Osteotome Sinus Elevation Groups. (**c**) Distribution of Perforations in Osteotomy in the Balloon and Osteotome Sinus Elevation Groups. (**d**) Distribution of Perforations in Elevation with Osteotomes in the Balloon and Osteotome Sinus Elevation Groups. (**e**) Distribution of Elevation of All Layers with Osteotomes in the Balloon and Osteotome Sinus Elevation Groups.

Table 3 and Figure 2b illustrate the distance of elevation from the crest measured with CBCT in the groups with balloon sinus elevation and osteotome elevation. The distance was greater in the balloon elevation group (*p* < 0.001), as analyzed using Student’s *t*-test. Normality test: Kolmogorov–Smirnov.

### 3.1. Perforation in Osteotomy

An exhaustive analysis was performed with Fisher’s Exact Test where the perforations produced with both techniques were compared both at the time of accessing the maxillary sinus and when membrane elevations were performed (see Table 4).

### 3.2. Perforation in Elevation with Osteotomes

There was significant difference in the comparison between the groups, there were more perforations in the osteotome sinus elevation group, Fisher’s Exact Test *p* ≤ 0.050 (see Table 5).

### 3.3. Elevation of All Layers with Osteotomes

There was no significant difference in the elevation of all layers between the groups, in 60% (n = 12) of the cases all layers were elevated, there was no elevation in 40% of the total cases (n = 8), Fisher’s Exact Test *p* ≤ 0.050 (see Table 6).

### 3.4. Multivariate Logistic Regression Analysis in the Balloon Technique Group

#### Dependent Variable: Elevation Distance (CBCT)

A logistic regression analysis was conducted to assess the relationship between the studied variables and the elevation distance using the balloon technique. Table 7 presents the omnibus test (*p* = 0.013) with an R2R2 value of 0.519. The regression model predicts 100% of cases with an elevation distance less than or equal to 10.65 mm and 100% of cases with an elevation distance greater than 10.65 mm. The Table 8, also shows the significant variables.

### 3.5. Multivariate Logistic Regression Analysis in the Osteotome Technique Group

#### Dependent Variable: Elevation Distance (CBCT)

A logistic regression analysis was conducted to assess the relationship between the studied variables and the elevation distance using the osteotome technique. Table 9 presents the omnibus test (*p* = 0.004) with an R2R2 value of 11.251. The regression model predicts 100% of cases with an elevation distance less than or equal to 9.60 mm and 100% of cases with an elevation distance greater than 9.60 mm. The Table 10 also shows the significant variables.

**Figure 3:**
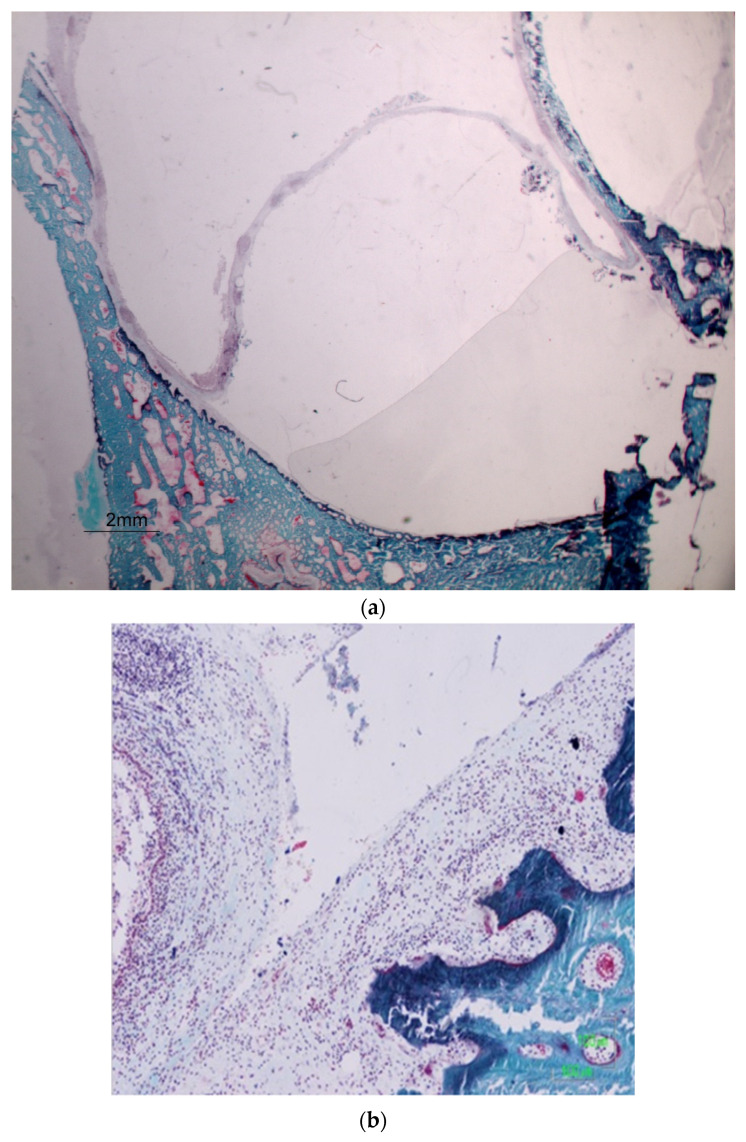

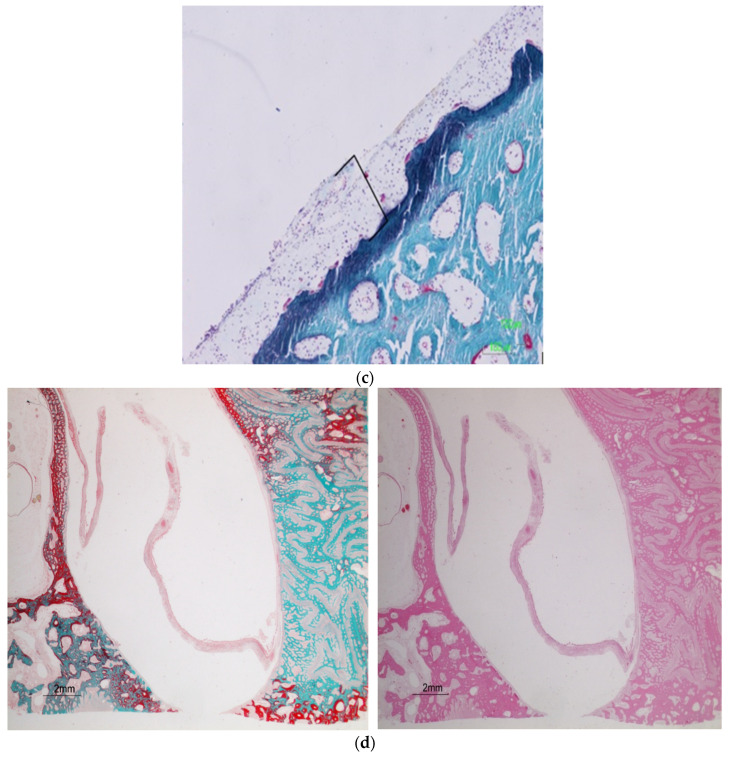

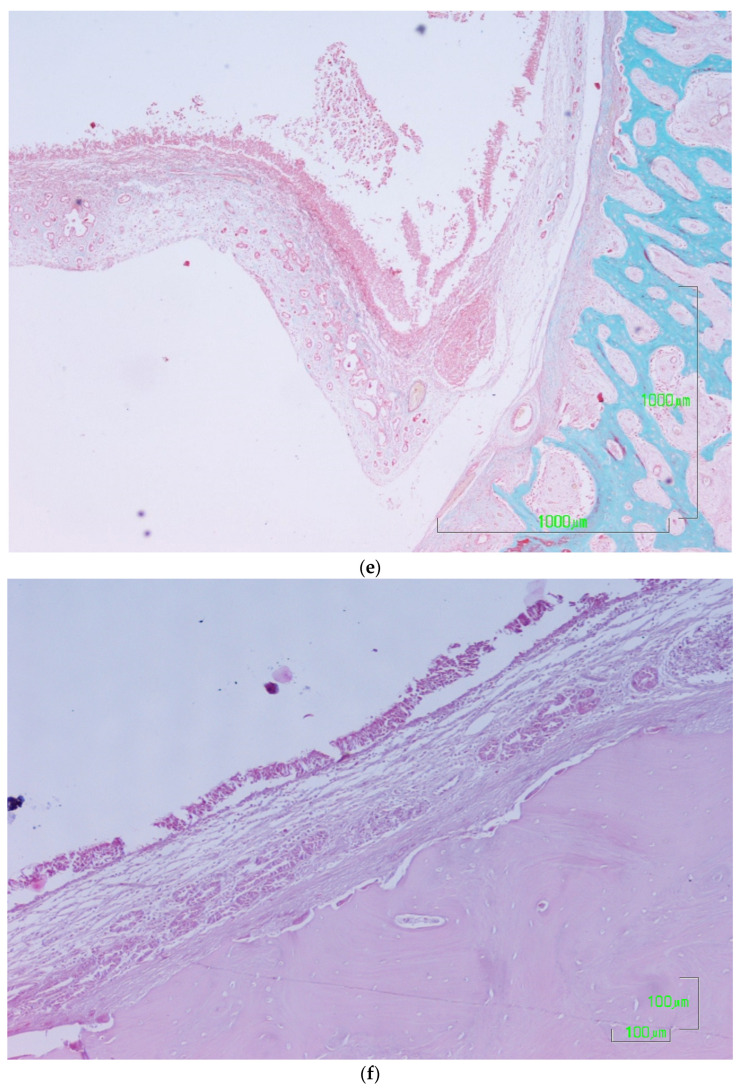
(a) Osteotomy, Elevated Mucosa at 100 µm Magnification. (**b**) Starting Zone of Elevation at 100 µm Magnification. (**c**) Periosteum at 100 µm Magnification. (**d**) Discontinuous Mucosal Elevation and Its Rupture. (**e**) Loss of Tissue Integrity. (**f**) Non-elevated Mucosal Area. The glandular layer is observed in the area closest to the beginning of the mucosal elevation.

The microscopic images of the histological samples did not reveal any tears or lacerations of the membranes in any of the samples analyzed with the balloon device. In only four cases was it not possible to elevate all layers, with the glandular layer remaining unraised. A fine layer of collagen tissue was observed in the bone, which could be interpreted as the periosteum or parts of it (see Figure 3a–c).

In this study, it was determined that the Osteotome Sinus Floor Elevation (OSFE) technique caused mucosal tears. These findings support several studies advocating for limitations on the height of elevation achievable with these methods. Perforations primarily occurred during the elevation process, with the majority of pressure being applied by the osteotome in the central area of the elevated mucosa (see Figure 3d–f).

## 4. Discussion

The preservation of the integrity of the sinus floor membrane is essential for ensuring the success of sinus elevation procedures. It has been documented that excessive stretching of this membrane can cause tears, particularly in cases involving significant elevations using osteotomes [25]. Techniques that distribute force over a larger area of the membrane, avoiding concentrated loads, have shown lower rates of mucosal lacerations [26,27].

The Schneider’s membrane perforation is the most common complication, especially in procedures with a lateral window, with a reported percentage of 35% according to Jensen et al. [28]. Similar results were obtained by Diaz Olivares et al. in their systematic review and meta-analysis, placing it at 30.6% of a total of 1598 surgeries performed [29]. This is the reason why there is a constant search for techniques that decrease the number of the same, as shown in the present study.

The osteotome technique, originally described by Tatum in 1994, has demonstrated the ability to achieve elevations of up to 5 mm without causing membrane perforations [30]. Therefore, a transalveolar approach that allows for the safe elevation of the membrane to heights greater than 5 mm is crucial. Tatum later modified his osteotome technique to include the insertion of bone particles into the sinus, which helps to avoid direct contact between instruments and the membrane [31]. Recently, various alternative methods to the osteotome technique have been proposed, including the use of inflatable devices like balloons or hydraulic pressure. These methods have demonstrated a reduction in membrane perforation rates [32,33,34,35].

The use of a balloon device, as described by Soltan and Smiler, has proven to be a highly successful and easy-to-use procedure. In this study, it was found that the osteotome sinus floor elevation technique (OSFE) caused mucosal tears [14]. These findings support other studies that propose limitations on the heights that can be achieved with this method [3,4,7]. Perforations mainly occurred during the elevation process, localized at the center of the elevated mucosa, where the osteotome applied the greatest pressure. In the perforated areas, the mucosa was significantly thinner compared to the undamaged mucosa elevated with the balloon device. Additionally, the mucosal elevation distance from the bone was smaller with OSFE compared to the balloon. It is presumed that uneven tension during mucosal detachment leads to over-expansion and subsequent tissue laceration [17,36,37].

The new balloon method achieved elevations without perforations in all analyzed cases, which is consistent with successful studies where membranes were elevated 8 to 10 mm in ex vivo human specimens fixed with formaldehyde [15,38].

Another finding was that the elevation of the sinus floor mucosal layers was not uniform with either method. OSFE demonstrated the complete elevation of the soft tissue, including the periosteum. This result is consistent with previous studies conducted by Summer Rosen et al. and Ardekian et al. [4,8,38].

On the other hand, the balloon method resulted in a division of the mucosa, leaving a thin layer of collagen tissue over the bone, which can be interpreted as the periosteum or part of it. The rest of the mucosa was elevated, including a thin layer of collagen tissue at the lower layer. These findings support previous studies on sinus floor elevation in human cadavers, where a division of the sinus floor mucosa into a layer of collagen tissue above the periosteum was observed. This layer has been anatomically described as the “reticular layer” or “locus minoris resistentiae” [19,39]. The collagen tissue near the periosteum contains osteoblast progenitor cells and, therefore, has osteogenic potential [40].

The pig head is a well-established animal model in dental implantology research due to its similarity to human anatomy. However, it is known that the mucosa of the maxillary sinus of the pig is thicker than that of humans. This can lead to higher adhesion and elastic tensile forces. Additionally, in pigs, there is a greater number of septa [30,41]. Despite these limitations and the sample size, the preliminary results of this study of favorable results are in favor of the new elevation device. The potential transfer of this procedure to a clinical environment has, therefore, been demonstrated, along with a demonstration of the general applicability of this technical configuration for future clinical trials.

## 5. Conclusions

The OSFE technique caused perforations in the sinus membrane at greater heights, whereas the new balloon device successfully elevated the membrane without rupturing it. OSFE was effective in completely lifting the soft tissue from the underlying bone, including the periosteum. In contrast, the balloon method was able to separate the mucosa into a clearly defined layer of tissue, leaving a thin layer of soft tissue above the bone. Given the limitations of this study, the use of the new balloon device and technique for sinus augmentation proved to be a minimally invasive procedure with favorable outcomes.

## 6. Patents

The sinus floor elevation was performed using the author’s patented new elevation control system, with international publication number WO/2022/200650 and PCT/ES2022/000008 in PATENTSCOPE. This system consists of a syringe containing stops, retaining elements, latex and serum.

## Figures and Tables

**Table 2:** Study of elevation lengths measured with CBCT.

**Sinus Lift with Balloon**	**Elevation Distance from Crest Measured with CBCT (mm)**	**Perforation in Osteotomy (Yes/No)**	**Perforation in Elevation (Yes/No)**	**Elevation of All Layers (Yes/No)**
1	10.6	no	no	yes
2	11	no	no	no
3	10.1	no	no	yes
4	10.7	no	no	yes
5	10.1	no	no	yes
6	12.5	no	no	no
7	9.8	no	no	no
8	13.3	no	no	yes
9	10.1	no	no	no
10	11.8	no	no	yes
**Sinus Lift with Osteotome**	**Elevation Distance from Crest Measured with CBCT (mm)**	**Perforation in Osteotomy (Yes/No)**	**Perforation in Elevation with Osteotomes (Yes/No)**	**Elevation of All Layers with Osteotomes (Yes/No)**
11	9.6	no	yes	yes
12	9.8	no	yes	yes
13	8	no	yes	yes
14	8.8	no	yes	yes
15	10.3	no	yes	yes
16	9	no	yes	yes
17	9.2	no	yes	no
18	9.6	no	yes	no
19	10.4	no	yes	no
20	10.6	no	yes	no

**Table 3:** Distance of elevation from the crest measured with CBCT in the balloon and osteotome sinus elevation groups.

Group	Mean Distance (mm)	Mean Distance (mm)	Mean Distance (mm)	Mean Distance (mm)
Balloon Sinus Elevation	11.00	1.16	10.65	9.80–13.30
Osteotome Sinus Elevation	9.53	0.80	9.60	8.00–10.60

**Table 4:** Distribution and comparison of perforations in osteotomy in the balloon and osteotome sinus elevation groups (Fisher’s Exact Test).

Groups	Balloon Sinus Elevation, n = 10		Osteotome Sinus Elevation, n = 10		Total, n = 20		*p*-Value
	n	%	n	%	n	%	
Yes	0	0.00	0	0.00	0	0.00	1.0000
No	10	100.00	10	100.00	20	100.00	1.0000

**Table 5:** Distribution and comparison of perforations in elevation with osteotomes in the balloon and osteotome sinus elevation groups.

Groups	Balloon Sinus Elevation, n = 10		Osteotome Sinus Elevation, n = 10		Total, n = 20		*p*-Value
	n	%	n	%	n	%	
Yes	0	0.00	10	100.00	10	50.00	0.0001
No	10	100.00	0	0.00	10	50.00	0.0001

**Table 6:** Distribution and comparison of elevation of all layers with osteotomes in the balloon and osteotome sinus elevation groups.

Groups	Balloon Sinus Elevation, n = 10		Osteotome Sinus Elevation, n = 10		Total, n = 20		*p*-Value
	n	%	n	%	n	%	
**Yes**	6	60.00	6	60.00	12	60.00	1.0000
**No**	4	40.00	4	40.00	8	40.00	1.0000

**Table 7:** Logistic regression analysis of elevation distance using the balloon technique.

		Distance ≤ 10.65 mm	Distance > 10.65 mm	%	Chi-Square	Nagelkerke R-Squared	Omnibus Test *p*-Value
Elevation Distance with Balloon Technique	Distance ≤ 10.65 mm	5	0	100.00	8.630	0.519	0.013
	Distance > 10.65 mm	0	5	100.00			
	Total Count			100.00			

**Table 8:** Odds Ratio (OR), *p*-value and 95% Confidence Interval for variables in the balloon elevation group.

Variable	B (Beta Coefficient)	Wald Statistic	*p*-Value	Odds Ratio (OR)	95% CI Lower	95% CI Upper
Perforation in Osteotomy (yes)	−1.099	4.526	0.033	0.333	−0.995	1.100
Perforation in Elevation with Osteotomes (yes)	21.203	0.0001	0.999	16.048	0.0001	21.210
Elevation of All Layers with Osteotomes (yes)	0.0001	0.0001	1.000	1.000	0.080	12.557

**Table 9:** Logistic regression model with the dependent variable: elevation distance (CBCT) using the osteotome technique.

		Distance ≤ 9.60 mm	Distance > 9.60 mm	%	Chi-Square	Nagelkerke R^2^	Omnibus Test *p*-Value
Elevation Distance Using the Balloon Technique	Distance ≤ 9.60 mm	6	0	100.00	11.251	0.610	0.004
	Distance > 9.60 mm	0	4	100.00			
	Total Count			100.00			

**Table 10:** Odds Ratios (OR) and 95% Confidence Intervals for independent variables associated with the elevation distance using the osteotome technique.

Variable	B (Beta Coefficient)	Wald Statistic	*p*-Value	Odds Ratio (OR)	95% CI Lower	Variable
Perforation in Osteotomy (yes)	−0.693	0.866	0.423	0.50	−0.690	0.700
Perforation in Elevation with Osteotomes (yes)	21.658	0.0001	0.999	25.57	0.0001	21.700
Elevation of All Layers with Osteotomes (yes)	0.693	0.641	0.600	2.00	0.150	26.734

## Data Availability

The original contributions presented in the study are included in the article, further inquiries can be directed to the corresponding author.
